# Host Age and Denture Wearing Jointly Contribute to Oral Colonization with Intrinsically Azole-Resistant Yeasts in the Elderly

**DOI:** 10.3390/microorganisms9081627

**Published:** 2021-07-30

**Authors:** Klaus-Peter Wojak, Gertrud F. Ungermann, Ichsan Ichsan, Emilia Gomez-Molero, Klaus Jung, Michael Weig, Friedemann Nauck, Dirk Ziebolz, Yvonne Gräser, Roland Nau, Uwe Groß, Bernd Alt-Epping, Oliver Bader

**Affiliations:** 1 Institute for Medical Microbiology and Virology, University Medical Center Göttingen, 37075 Göttingen, Germany; kpwojak@web.de (K.-P.W.); flockeu@yahoo.de (G.F.U.); ichsanmd_aceh@yahoo.com (I.I.); emiliagomez803@hotmail.com (E.G.-M.); mweig@gwdg.de (M.W.); ugross@gwdg.de (U.G.); 2 Department of Palliative Medicine, University Medical Center Göttingen, 37075 Göttingen, Germany; friedemann.nauck@med.uni-goettingen.de (F.N.); bernd.alt-epping@med.uni-heidelberg.de (B.A.-E.); 3 Institute for Animal Breeding and Genetics, University of Veterinary Medicine Hannover Foundation, 30559 Hannover, Germany; Klaus.Jung@tiho-hannover.de; 4 Department of Cariology, Endodontology and Periodontology, University of Leipzig, 04103 Leipzig, Germany; Dirk.Ziebolz@medizin.uni-leipzig.de; 5 Institute for Microbiology and German National Consiliary Laboratory for Dermatophytes, University Medicine Berlin–Charité, 12203 Berlin, Germany; yvonne.graeser@charite.de; 6 Department of Geriatrics, Protestant Hospital Göttingen-Weende, 37075 Göttingen, Germany; rnau@gwdg.de

**Keywords:** *Candida glabrata*, oral colonization, aging, elderly, drug resistance, biofilm formation, denture

## Abstract

In elderly patients, several morbidities or medical treatments predisposing for fungal infections occur at a higher frequency, leading to high mortality and morbidity in this vulnerable patient group. Often, this is linked to an innately azole-resistant yeast species such as *Candida glabrata* or *C. krusei*. Additionally, host age per se and the wearing of dentures have been determined to influence the mix of colonizing species and, consequently, the species distribution of invasive fungal infections. Since both old age and the wearing of dentures are two tightly connected parameters, it is still unclear which of them is the main contributor. Here, we performed a cross-sectional study on a cohort (*N* = 274) derived from three groups of healthy elderly, diseased elderly, and healthy young controls. With increasing host age, the frequency of oral colonization by a non-*albicans* *Candida* species, mainly by *C. glabrata*, also increased, and the wearing of dentures predisposed for colonization by *C. glabrata* irrespectively of host age. Physically diseased hosts, on the other hand, were more frequently orally colonized by *C. albicans* than by other yeasts. For both *C. albicans* and *C. glabrata*, isolates from the oral cavity did not generally display an elevated biofilm formation capacity. In conclusion, intrinsically azole-drug-resistant, non-*albicans* *Candida* yeasts are more frequent in the oral cavities of the elderly, and fungal cells not contained in biofilms may predispose for subsequent systemic infection with these organisms. This warrants further exploration of diagnostic procedures, e.g., before undergoing elective abdominal surgery or when using indwelling devices on this patient group.

## 1. Introduction

Invasive fungal infections are a cause of high mortality and morbidity. Often, this is linked to inadequate antifungal therapy [1,2], which, at least partially, may be attributed to the increased incidence of innately azole-resistant yeast species such as *C. glabrata* or *C. krusei* [2,3]. Different fungal species, most prominently *C. albicans*, are already part of the commensal gut and skin microbiome in healthy humans. Clinical isolates of medically important *Candida* species often display intensive biofilm formation phenotypes on a variety of clinically used materials [4,5]. From there, they may disseminate into the host upon the deterioration or suppression of the immune system, microbiome imbalance, or simply upon the breaking of physical barriers [6].

In elderly patients, several morbidities (e.g., diabetes mellitus or cancer) or medical treatments (e.g., immuno-suppressive therapy, indwelling devices, or abdominal surgery) occur at a higher frequency, additionally predisposing this vulnerable group to invasive fungal infections. Therefore, in ageing societies, the diagnosis and treatment of fungal infections are gaining even more importance.

In addition to the gastrointestinal tract, the oral cavity has been determined to constitute a major reservoir of potential pathogenic microbes [7], including intrinsically azole-resistant yeast species such as *C. glabrata*.

Next to the classical predisposing factors for fungal infections, host age per se [8,9] and the wearing of dentures [10] or removable orthodontic appliances [11] have also been determined to influence the mix of colonizing species and thus the species distribution of invasive fungal infections [12]. Most *Candida* species are vivid formers of biofilms on various surfaces, including epithelia, silicon catheters, or simply polystyrol plastics. This includes *C. glabrata*, where the reference genome encodes at least seven families of adhesins or adhesin-like proteins [13] and clinical isolates show the further amplification of these families [14].

Since both old age and the wearing of dentures are two tightly interconnected parameters, it is still unclear which of them is the main contributor. In this study, we re-addressed these questions in cohorts of healthy and diseased elderly people and asked if these cohorts were independently associated with specific clinical parameters, host factors, or the biofilm formation capacity of yeasts on denture materials.

## 2. Materials and Methods

### 2.1. Study Cohort and Sampling

Our study cohort was designed to cover both physically healthy and diseased elderly people (here defined as 60 years of age and above) as well as a young control group (advanced medical students of the Georg-August-University of Göttingen, mean age 25 y, (Table 1)). The diseased elderly individuals were from three different hospital environments (intensive care units, palliative care units, and geriatric care units), and the healthy elderly individuals were from nursing homes in the vicinity. For the purpose of this study, the residents of nursing homes suffering from dementia were classified as physically healthy.

Each participating person was informed about the study’s content and purpose in accordance with the Helsinki declaration. Written consent was obtained before any samples were taken. In a minority of cases where patients were not able to consent themselves, consent was obtained from legal representatives. This study was approved by the ethical commission of the University Medical Center Göttingen (approval #9/10/09, October 2009).

Study participants were interviewed and given a short oral examination. Swabs (Transsystem, COPAN, Murrieta, CA, USA) were taken from (a) the oral cavity going over the cheek, palate, and tongue, (b) the inguinal fold, and (c) the interdigital area, without prior disinfection of the skin.

### 2.2. Fungal Differentiation and Drug Resistance Testing

The swabs were streaked onto Sabouraud and malt dextrose agar plate sets supplemented with chloramphenicol and gentamicin and cultivated at both 30 °C and 37 °C for up to two days. The culture-positive swabs were again streaked onto Chromagar (Oxoid) plates to additionally detect multiple species not apparent from the first culture. All isolates obtained were initially archived using the Cryobank system (Mast Diagnostica). Isolate species were identified by MALDI-TOF MS (MALDI BioTyper database version 2.1.0.1, Bruker Daltonics, Bremen, Germany). Multiple colonies of the same species that were recovered from the same sample were counted as only one isolate. Antifungal drug susceptibilities towards fluconazole, voriconazole, micafungin, nystatin, and amphotericin B were determined according to the EUCAST EDef 7.1 method [15].

Four isolates, which could not be regrown from cryostocks, were differentiated by the sequencing of the ITS2 locus [16] amplified from DNA prepared directly from the ceramic beads. Subsequently, they were omitted from further resistance testing. Two further isolates of *S. cerevisiae* that failed to grow in the AM-3 medium were omitted from the polyene resistance testing, and one and four isolates of *S. cerevisiae* and *C. albicans* that failed to grow in the RPMI medium were omitted from the azole resistance testing and echinocandin resistance testing, respectively.

### 2.3. Dermatophyte Sampling and Differentiation

To screen for dermatophytes in the interdigital and inguinal fold swabs, one additional plate set was prepared as described above and incubated at 26 °C for up to six weeks. No screening for dermatophytes was conducted with oral swabs. The species of the resulting non-yeast colonies were differentiated by the sequencing of the ITS 1/2 locus of the 35S rDNA gene [17].

### 2.4. Biofilm Formation on Denture Material

To determine the capacity to form biofilms of the individual *C. albicans* and *C. glabrata* isolates, custom-made polished 5 × 5 × 1 mm acrylic ester denture tester pieces were used (Aesthetic Autopolymerisat, Candulor AG, Pinten, Switzerland). The fungal isolates were cultured on Sabouraud agar at 30 °C overnight and diluted to an OD of 0.8 McFarland in 0.7% NaCl. In total, 100 µL of the cell suspension was added to 2 mL of YPD (1% yeast extract, 1% peptone, and 2% glucose) in a glass tube containing a single tester piece and incubated at gentle shaking at 37 °C overnight. For the removal of planktonic cells, the tester pieces were thoroughly but carefully flushed with PBS. Subsequently, the tester pieces were placed in 1.5 mL of PBS in 12-well cell culture plates, and the attached cells were scraped off the material manually and suspended by pipetting. For the semi-quantification of the attached biofilm, the optical density of the resulting cell suspension was determined spectrophotometrically at 600 nm. All values were calculated as averages of three biologically independent experiments.

### 2.5. Statistical Data Analysis

All analyses were first conducted for the whole study collective and then separately for the control—the nursing home residents and inpatients group. Age was described by its mean +/− standard deviation; categorical parameters were described by absolute and relative frequencies. The study parameters were compared univariately between patients with and without symptoms by Student’s *t*-test (age) and by Fisher’s exact test (categorical parameters) and further described by odds ratios where adequate. Additionally, for each symptom, a multiple logistic regression model was fitted (including specified interaction terms of interest) with subsequent stepwise variable selection using Akaike’s information criterion. The resulting models were only reported if more than one study parameter survived the selection procedure. The significance level was set to α = 5%. All analyses were performed using the software R (version 3.1.2., www.r-project.org, accessed on 31 Octorber 2014).

## 3. Results

### 3.1. Cohort

We were able to recruit a total of 274 individuals into our study (Table 1). The initial goal was to recruit 50 individuals per subgroup; nursing home #3 was therefore taken up into the study to augment recruitment into the nursing home category, where these numbers could not be reached initially. The age distribution within the target range of 60 years and above was shifted by approximately 10 years between the diseased and healthy elderly groups, peaking in the seventh and eighth decade of life, respectively (Table 1).

### 3.2. Fungal Colonization

**Figure 1:**
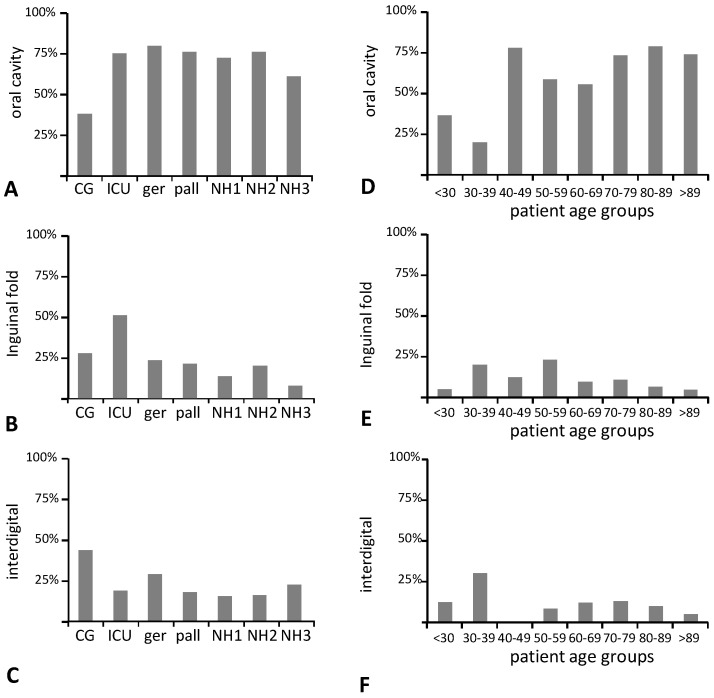
Mean rates of fungal colonization. (**A**–**C**) stratified by subcohort; (**D**–**F**) stratified by patient age group. (**A**,**D**) oral cavity; (**B**,**E**) inguinal fold; (**C**,**F**) interdigital; CG, control group; ICU, intensive care unit; ger, geriatric care unit; pall, palliative care unit; NH1-3, nursing homes 1-3.

The rates of fungal colonization were analyzed at three different body sites. In the oral cavity, colonization rates were increased to 60–80% in all study subgroups as compared to the young control group (38%), independent of the ‘diseased’ or ‘healthy’ statuses (Figure 1A). In contrast, the colonization of the inguinal fold was highest in the group of ICU patients (51% as compared to 8–28% in all other subgroups, Figure 1B). Interdigital colonization was highest in the control group (45% as compared to 16–35% in all others, Figure 1C). When stratified by age, oral colonization showed an overall moderate correlation with age groups, with older individuals being more frequently colonized than younger individuals (Figure 1D). In samples from the inguinal fold or interdigital region, this was not evident (Figure 1E,F).

The species differentiation of the 284 yeast isolates obtained showed a vast majority of *Candida albicans* (*n* = 140; 49.3%) and *Candida glabrata* (*n* = 96; 33.8%), followed by *Candida parapsilosis* (*n* = 15; 5.3%), *Candida tropicalis* (*n* = 10; 3.5%), *Pichia guilliermondii* (*n* = 7; 2.5%), and *Candida krusei* (*n* = 5; 1.8%). Other yeast species (*n* = 13 isolates; 4.7%) isolated with low frequency were *Candida bracarensis* (*n* = 1), *Candida dubliniensis* (*n* = 2), *Candida famata* (*n* = 1), *Candida kefyr* (*n* = 2), *Candida metapsilosis* (*n* = 1), *Pichia norvegensis* (*n* = 1), and *Saccharomyces cerevisiae* (*n* = 3). From oral specimens, mixed cultures were obtained in 29 (15.6%) cases (Table 2).

There were only six cases of dermatophytes (here: *Trichophyton rubrum*), all isolated from interdigital swabs (2.5%). Comparable studies have shown a prevalence of toenail onychomycosis of 3.2% in the general population and 10.3% in the elderly population (61 years and up) [18]. Other molds growing after long-term incubation from interdigital and inguinal fold samples were *Aspergillus sydowii*, *Eutypella prunastri*, *Lewia infectoria*, *Merismodes fasciculate*, *Penicillium citreonigrum*, *Penicillium daleae*, *Penicillium expansum*, *Penicillium lanosum*, and *Thanatephorus cucumeris*. Since clinical symptoms were absent, these were considered of no further clinical relevance.

### 3.3. Antifungal Drug Susceptibility

With the exception of two azole-resistant *C. albicans* (MIC_50_ values = 8 [FLZ]; 0.125 and 0.250 [VRZ]) and one azole-resistant *C. parapsilosis* isolate (MIC_50_ values = 8 [FLZ] and 2 [VRZ]), the MIC values for these drugs were within the reported ranges for clinically susceptible isolates (Supplementary Figure S1). No reduced susceptibilities were observed for polyenes (Amphotericin B and Nystatin). Azole resistance (FLZ and VRZ) was generally observed in *C. glabrata* and *C. krusei* (Supplementary Figure S1C,D) and elevated echinocandin (Micafungin) MIC values in *C. parapsilosis sensu lato* isolates (Supplementary Figure S1E).

### 3.4. Increasing Host Age and Wearing of Dentures Are Both Predictors of Oral Colonization with C. glabrata

**Figure 2:**
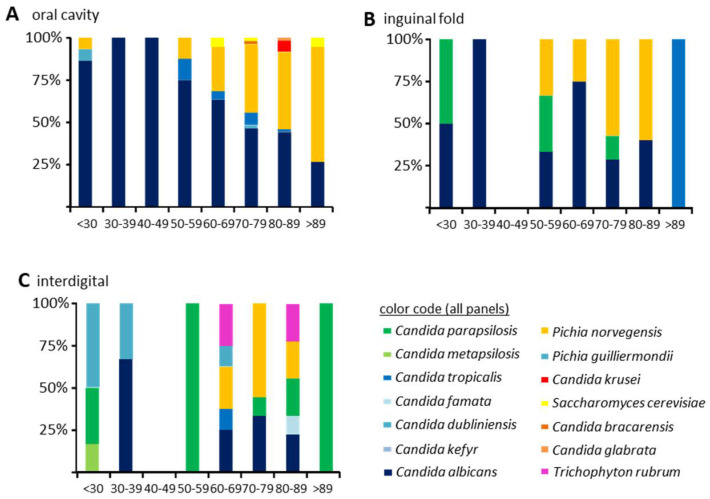
Species stratified by host age. The percentage of individuals colonized in different age groups is given. Oral (**A**), inguinal fold (**B**), and interdigital (**C**) specimens. Color code corresponds to the one used in Supplementary Figure S1.

An analysis of colonization with respect to fungal species and host age confirmed an increasing oral prevalence of non-*albicans Candida* species with increasing age (p_mult_ = 0.0006, Table 3). This was dominated by *C. glabrata* (Figure 2A), but other species of the Nakaseomyces clade (*C. bracarensis*), as well as *S. cerevisiae* and *C. krusei,* were also present only at increased host ages (Figure 2A). In inguinal fold or interdigital specimens, the differences of the age-dependent colonization were not statistically significant (*p* = 0.9892 and *p* = 0.1109, respectively). *C. glabrata* was not observed in sufficient numbers outside the oral cavity to allow for statistical comparisons (Supplementary Tables S1 and S2).

**Figure 3:**
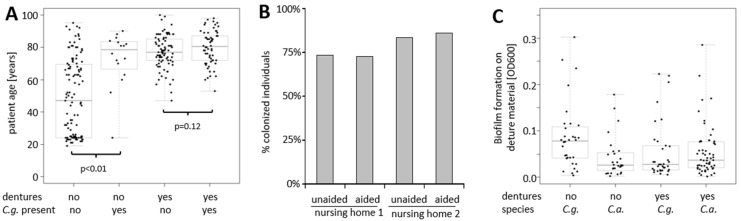
Factors potentially influencing oral colonization. (**A**) The influence of denture wearing and host age on colonization by *C. glabrata*. (**B**) The influence of receiving aid for dental cleaning on fungal colonization, and (**C**) the correlation of biofilm formation capacity of oral *C. albicans* and *C. glabrata* isolates on acrylic ester between non-denture wearers vs. denture wearers.

Among carriers of *C. glabrata*, denture wearers were highly dominant (Figure 3A columns 2 vs. 4, and Table 2). The multiple logistic regression model with age, study group, yeast carriage, and presence of dentures as explaining variables did not confirm statistical significance (p_mult_ = 0.1243) for dentures across the entire collective, but the absence of a statistically significant age-dependency among denture wearers (Figure 3A, columns 3 and 4) suggests a positive effect of denture wearing for colonization with *C. glabrata*, potentially overriding the age-dependency. The presence of yeasts in the oral cavity was also independent of whether individuals were able or unable to clean their teeth or dentures without assistance (*p* = 0.1461, Table 3), exemplified by nursing homes 1 and 2 (Figure 3B).

All *C. glabrata*, as well as a similar number of oral *C. albicans* isolates from this study, were tested for their capacity to form biofilms on acrylic ester denture material (Figure 3C). While we were able to identify several isolates of both species with increased biofilm formation capacity, there were no statistically significant (logistic regression: *p* = 0.12) differences between isolates obtained from denture-wearers vs. non-denture-wearers.

### 3.5. Oral Colonization with C. albicans Is Different between Healthy and Diseased Subjects

An analysis of oral colonization with respect to yeast species and host health status (Table 3) revealed a strong correlation of *C. albicans* with the inpatient group (*p*_mult_ = 0.0007). While diseased subjects were more likely to be colonized by *C. albicans* (51%), healthy elderly subjects (living in nursing homes) were colonized only to the same degree (28%) as the young control group (28%). In contrast, colonization by other species was higher in healthy subjects (51%) as compared to the diseased subjects (36%) and the young control group (4%).

## 4. Discussion

Old age and denture-wearing are highly correlated parameters. It is, therefore, intrinsically difficult to judge their respective contributions to oral yeast colonization. Our data show that both factors contribute independently: the influence of host age is also present in the absence of dentures, and among individuals colonized by *C. glabrata*, denture wearers are more common than they are among those colonized by *C. albicans*. These findings are in line with several previous reports looking at individual parameters correlating with oral colonization by different yeast species [8,9,10,19]. We also did not find differences in colonization dependent on oral hygiene, which is supported by others [20], or an age-dependent increase in the general fungal colonization of surfaces, as exemplified by probing the inguinal fold and interdigital space.

*C. albicans* is the most prevalent yeast involved in invasive fungal disease and several varieties of oral lesions, including oral cancer [21]. Several predisposing factors are known, most importantly the suppression or deterioration of the immune system. Even though none of the individuals enrolled in this study showed specific signs of an oral fungal disease such as thrush, it was not surprising to see this species enriched among hospitalized patients, where general predisposition would be increased. A specific association of *C. albicans* with patient age, as reported before [8,9,22], or denture wearing [23] was not, however, confirmed in our cohort.

In contrast, our study highlights *C. glabrata* as an oral colonizer in the elderly. What are potential host factors that could underlie the observed species shift towards non-*albicans Candida* species and *C. glabrata* in particular? Among many changes associated with host ageing, the wearing of dentures and modulations of innate and acquired immunity over time have been discussed most intensively.

The process of immune system ageing, referred to as immunosenescence [24], indeed impacts the Th-17 cell population, the main mucosal defense system against fungi [25]. In contrast, dendritic cells (relying more on TLR2/4 mediated signaling) do not appear to lose their potency against *C. albicans* with increased host age [26]. The increased occurrence of non-*albicans Candida* species is also seen in patients with advanced cancer [27], which might be similarly correlated with changes in cytokine regulation and skewed T-cell, including Th-17 cell, populations [28,29]. Mucosal Th-17 cell activation occurs via a cascade initiated by dectin-1 [30] recognizing free β-1,3-glucan, a major constituent of most fungal cell walls. Although comparatively little is known about the specific immune recognition of *C. glabrata*, *C. albicans* and *C. glabrata* are apparently recognized very differently by the immune system [31]. For example, while *C. albicans* can principally evade macrophages [32], *C. glabrata* may even propagate inside the phagosome. Uptake into macrophages is more dependent on mannan than on glucan [33]. Indeed, as compared to *C. albicans*, the glucan content of *C. glabrata* cell walls is reduced, while mannan is increased [34]. Therefore, the correlation of host age with *C. glabrata* colonization may point to age-associated shifts in the macrophage population towards less receptive types or to simple quantitative changes in immune cell abundances.

The yeast species composition of the oral swabs of the wearers is representative of that seen in biofilms on the respective worn dentures [10]. *C. glabrata* is unique among the *Candida* yeasts with respect to its highly increased inventory of genes coding for adhesins and adhesin-like proteins [13,34]. Most of these remain functionally uncharacterized and may well also contribute to biofilm formation on denture acrylics. Counterintuitively, only a minority of *C. albicans* and *C. glabrata* isolates from our study actually showed an increase in vitro biofilm formation on the acrylic ester. This phenomenon has also been seen by others [19]. Hypothetically, this may indicate that the expression of the—yet to be identified—relevant factors for forming biofilms on such materials is highly variable within a clonal population, as seen for the *C. glabrata* lectin Epa1 [35], and that those cells obtained from oral swabs were shed from denture biofilms due to their lack of respective adhesin expression.

Such dynamic processes could substantially contribute to a permanent adaptation of the yeast cells to the different environmental requirements necessary for successful colonization.

Alternatively, the formation of biofilms on dentures might not be a contributing factor themselves, but rather the local environment generated by the wearing of dentures could potentially affect saliva flow and its composition, forming an environment more suitable for planktonic *Candida* growth. Lastly, it could even be hypothesized that the regular cleaning of dentures counter-selected for the presence of highly biofilm-forming organisms, as these would be regularly removed from the oral cavity.

Although *C. albicans* is still the key organism, intrinsically azole-drug-resistant, non-*albicans Candida* yeasts are more frequent in the oral cavity of the elderly and even more so in the oral cavity of denture wearers. Such oral colonization with opportunistic yeast pathogens may predispose for the subsequent infection of these specific organisms. This is highlighted by a similar epidemiologic species–age relation in samples obtained from primary sterile sites, such as blood cultures [12].

## 5. Conclusions

Our findings may alter the awareness of non-*albicans Candida* species and their spectrum of therapeutic resistance in the general care of elderly people. Due to *C. glabrata* increasingly gaining importance in several patient groups, such as abdominal surgery patients, or due to the occurrence of echinocandin resistance in this species [36,37,38], our findings should be taken into account when treating elderly patients. Screening for oral [39] or gastrointestinal fungal colonization prior to surgical intervention or when employing indwelling devices might be a tool to facilitate adequate subsequent calculated antifungal therapy [1].

## Figures and Tables

**Table 1:** Cohort description.

Study Group	Health Status	Total	Mean (+/− SD) Age	Male/Female
control group	healthy	46	25 ± 3.6 y	67%/33%
palliative care unit	diseased	50	65 ± 10.0 y	34%/66%
intensive care unit	diseased	47	67 ± 15.6 y	59%/41%
geriatric care unit	diseased	51	80 ± 6.7 y	44%/56%
nursing home #1	healthy	37	83 ± 8.1 y	81%/19%
nursing home #2	healthy	30	86 ± 8.6 y	67%/33%
nursing home #3	healthy	13	81 ± 13.1 y	54%/46%

**Table 2:** Culture composition from the oral specimen.

Yeast Species	Dentures	
	No	Yes
(culture negative)	81	115
*C. albicans* only	54	38
*C. albicans* + other yeast	2	4
*C. albicans* + *C. glabrata*	4	14
*C. albicans* + *C. glabrata* + other yeast(s)		2
*C. glabrata* only	13	41
*C. glabrata* + other yeast(s)	1	2
other yeast(s) only	7	4
total culture-positive specimen	81	105
% culture positivity	50%	48%
% positive containing *C. albicans*	74%	50%
% positive containing *C. glabrata*	31%	55%

**Table 3:** Patient characteristics and correlations with yeast colonization of the oral cavity.

Parameter	Level	Presence of *C. albicans*				Presence of Non-*albicans Candida* Species				Presence of *C. glabrata*			
		No	Yes	OR	*p*, (*p*_mult_)	No	Yes	OR	*p*, (*p*_mult_)	No	Yes	OR	*p*, (*p*_mult_)
age	years	65.53 +/− 23.84	66.14 +/− 20.68		0.8227 (0.0908)	59.81 +/− 24.04	76.82 +/− 14.07		<0.0001 (0.0006)	60.75 +/− 23.76	78.40 +/− 12.33		<0.0001 (0.0010)
gender	female	96(62%)	60(38%)	1.22	0.4572	98(63%)	58(37%)	0.80	0.4435	108(69%)	48(31%)	0.77	0.3473
	male	67(57%)	51(43%)			80(68%)	38(32%)			88(75%)	30(25%)		
health status	control	33(72%)	13(28%)		<0.0001 (0.0007)	44(96%)	2(4%)		<0.0001 (0.0628)	46(100%)	0(0%)		<0.0001 (0.0189)
	healthy	58(72%)	22(28%)			39(49%)	41(51%)			45(56%)	35(44%)		
	diseased	72(49%)	76(51%)			95(64%)	53(36%)			105(71%)	43(29%)		
antibacterials	no	136(61%)	87(39%)	1.39	0.3432	147(66%)	76(34%)	1.25	0.5172	164(74%)	59(26%)	1.65	0.1259
	yes	27(53%)	24(47%)			31(61%)	20(39%)			32(63%)	19(37%)		
antimycotics	no	156(60%)	104(40%)	1.50	0.5781	166(64%)	94(36%)	0.30	0.1485 (0.9856)	183(70%)	77(30%)	0.18	0.1234 (0.9942)
	yes	7(50%)	7(50%)			12(86%)	2(14%)			13(93%)	1(7%)		
independent oral hygiene	yes	125(61%)	79(39%)	1.33	0.3254	138(68%)	66(32%)	1.56	0.1461	152(75%)	52(25%)	1.72	0.0672
	no	38(54%)	32(46%)			40(57%)	30(43%)			44(63%)	26(37%)		
oral dentures present	no	80(61%)	51(39%)	1.13	0.6243 (0.9167)	107(82%)	24(18%)	4.49	<0.0001 (0.2331)	115(88%)	16(12%)	1.47	<0.0001 (0.1243)
	yes	83(58%)	60(42%)			71(50%)	72(50%)			81(57%)	62(43%)		
health status X age									(0.0185)				(0.0055)
health status X antimycotics									(0.9851)				(0.9932)
health status X dentures									(0.0219)				(0.0243)
age X dentures					(0.9300)				(0.8903)				(0.4091)

OR: odds ratio.

## Supplementary Materials

The following are available online at https://www.mdpi.com/article/10.3390/microorganisms9081627/s1. Figure S1: Yeast antifungal drug susceptibility; Table S1: Patient characteristics and correlations with yeast colonization of the inguinal fold; Table S2: Patient characteristics and correlations with yeast colonization of the interdigital space.
