# Supplementary material for: Host Age and Denture Wearing Jointly Contribute to Oral Colonization with Intrinsically Azole-Resistant Yeasts in the Elderly
Source: Microorganisms. 2021 Jul 30;9(8):1627. doi: 10.3390/microorganisms9081627 (PMC8400291; doi:10.3390/microorganisms9081627)
Supplement: Supplementary file 1 [file microorganisms-09-01627-s001.zip › 2021 MO - elderly -Supp_data_V1.pdf]

Supplementary data for:

Host age and denture wearing jointly contribute to oral colonization with innately azole-resistant yeasts in the elderly

Klaus-Peter Wojak[1,2*], Gertrud F. Ungermann[1,2*], Ichsan[1], Emilia Gomez-Molero[1], Klaus Jung[3], Michael Weig[1], Friedemann Nauck[2], Dirk Ziebolz[4&], Yvonne Gräser[5], Uwe Groß[1], Bernd Alt-Epping[2§], and Oliver Bader[1§$]

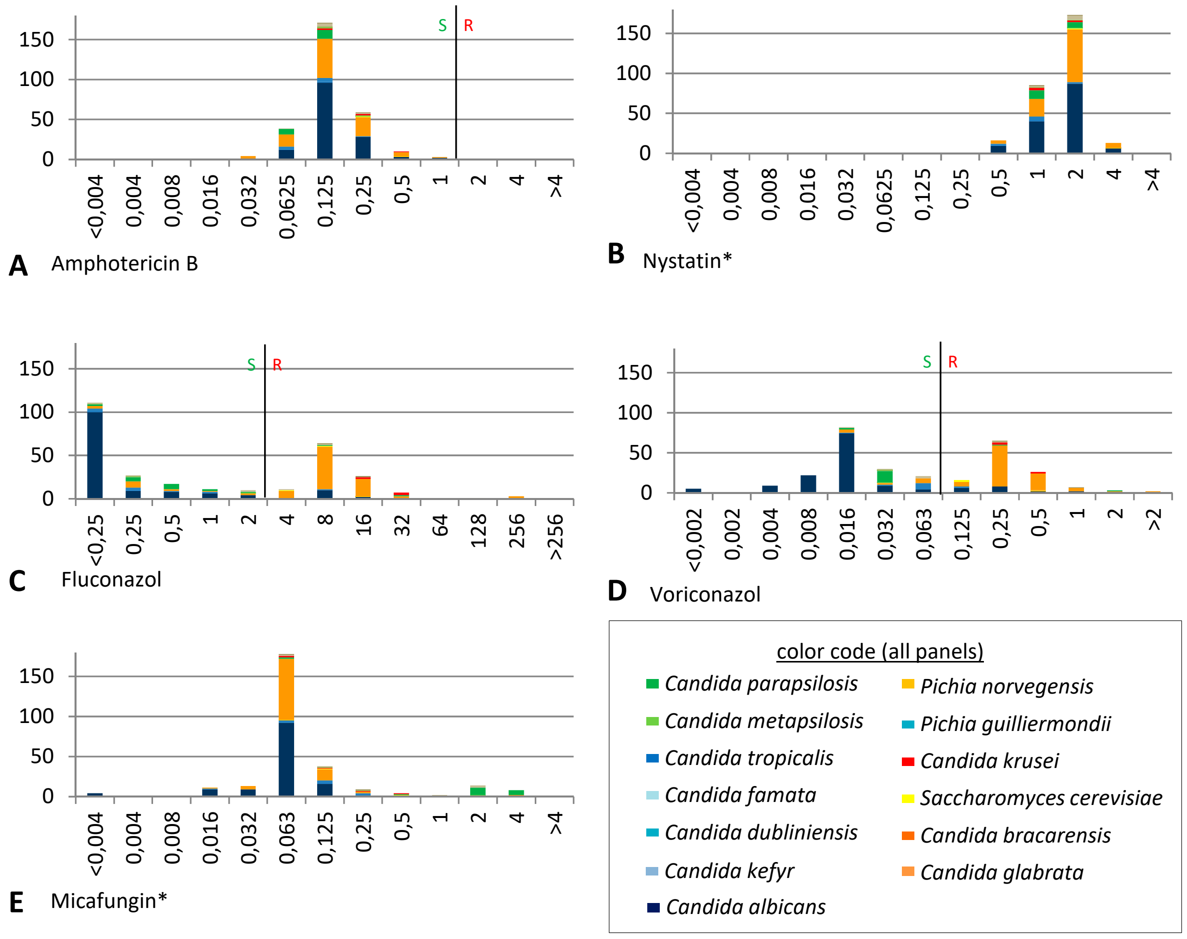

Supplementary Figure S1: Yeast antifungal drug susceptibility. (A-E) Antifungal drug susceptibilities according to EUCAST broth micro dilution, stratified by yeast species. x-axis: MIC_{80} (Amphotericin B) or MIC_{50} values (all others); y-axis: number of isolates. Color code for species corresponds to the one used in Figure2. [a] no clinical breakpoints available for either Micafungin nor Nystatin.

Supplementary Table S1: Patient characteristics and correlations with yeast colonization of the inguinal fold.

parameter	level	presence of *C. albicans* [a]				presence of non-*C. albicans* species [a]			
		**no**	**yes**	**OR**	**p**	**no**	**yes**	**OR**	**p**
age	years	65,96 +/-22,74	61,75 +/-19,03		0,4718	65,78 +/-22,71	65,71 +/-21,00		0,9892
gender	female	153(98%)	3(2%)	4,19	0,0338	150(96%)	6(4%)	2,56	0,0778
	male	109(92%)	9(8%)			107(91%)	11(9%)		
health status	control	44(96%)	2(4%)		0,0590	43(93%)	3(7%)		0,2446
	healthy	80(100%)	0(0%)			78(98%)	2(2%)		
	diseased	138(93%)	10(7%)			136(92%)	12(8%)		
anti-bacterials	no	216(97%)	7(3%)	3,33	0,0515	211(95%)	12(5%)	1,91	0,3292
	yes	46(90%)	5(10%)			46(90%)	5(10%)		
anti-mycotics	no	250(96%)	10(4%)	4,13	0,1193	244(94%)	16(6%)	1,17	0,6013
	yes	12(86%)	2(14%)			13(93%)	1(7%)		

OR: odds ratio.

[a] None of the parameters survived stepwise variable selection from the multivariate logistic model.

Supplementary Table S2: Patient characteristics and correlations with yeast colonization of the interdigital space.

parameter	level	presence of *C. albicans*				presence of non-*C. albicans* species [a]			
		**no**	**yes**	**OR**	**p, (pmult)**	**no**	**yes**	**OR**	**p**
age	years	65,89+/-22,65	62,6+/-21,44		0,6446	66,49+/-22,49	58,68+/-22,64		0,1109
gender	female	153(98%)	3(2%)	3,20	0,1058 (0.2160)	149(96%)	7(4%)	3,81	**0,0027**
	male	111(94%)	7(6%)			100(85%)	18(15%)		
health status	control	44(96%)	2(4%)		0,3936 (0.9120)	39(85%)	7(15%)		0,0809
	healthy	79(99%)	1(1%)			77(96%)	3(4%)		
	diseased	141(95%)	7(5%)			133(90%)	15(10%)		
anti-bacterials	no	217(97%)	6(3%)	3,06	0,0938	203(91%)	20(9%)	1,10	0,7916
	yes	47(92%)	4(8%)			46(90%)	5(10%)		
anti-mycotics	no	250(96%)	10(4%)	0,00	1,0000 (1.0000)	237(91%)	23(9%)	1,71	0,3716
	yes	14(100%)	0(0%)			12(86%)	2(14%)		
health status x gender					(0.1010)				
health status x anti-mycotics					(1.0000)				

OR: odds ratio. Boldface: statistically significant correlations.

[a] None of the parameters survived stepwise variable selection from the multivariate logistic model.
