# Supplementary figures and images for: Host Age and Denture Wearing Jointly Contribute to Oral Colonization with Intrinsically Azole-Resistant Yeasts in the Elderly
Source: Microorganisms. 2021 Jul 30;9(8):1627. doi: 10.3390/microorganisms9081627 (PMC8400291; doi:10.3390/microorganisms9081627)

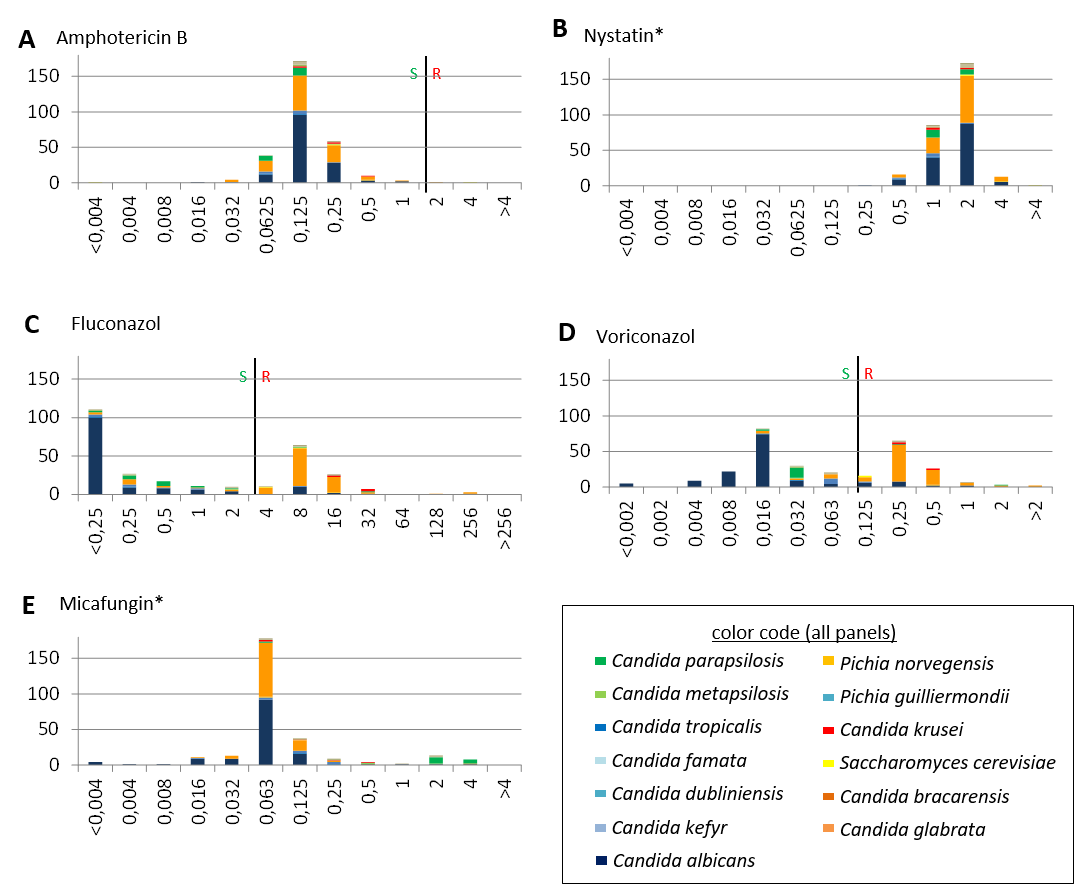

Supplement: Supplementary file 1 [file microorganisms-09-01627-s001.zip › SupFigs1.tif]
